# Supplementary material for: Incidence of Isolated Biliary Atresia during the COVID Lockdown in Europe: Results from a Collaborative Project by RARE-Liver
Source: J Clin Med. 2023 Jan 18;12(3):775. doi: 10.3390/jcm12030775 (PMC9918263; doi:10.3390/jcm12030775)
Supplement: Supplementary file 1 [file jcm-12-00775-s001.zip › jcm-2127425-supplementary.pdf]

Table S1: Spearman's correlation coefficient between the percentage change in IBA patients born per month and stringency index per country.

Centres	Correlation coefficient	*p*-value
All	-0.33	0.31
Hannover excluded	-0.74	0.02
Hannover and Padova excluded	-0.73	0.03
National BA data only	-0.60	0.35

Table S2. Results from multivariate logistic regression analysis using IBA as a binominal dependent (outcome) variable. Stringency index was a continuous independent variable and country was a categorical independent variable. Odds ratio with 95% confidence intervals (CI) are shown. For the country variable, United Kingdom was used as a reference category.

Variable	Odds ratio (95% CI)	P-value	Total IBA patients (N)
Stringency index	0.99 (0.98 - 1.00)	0.25	
United Kingdom	ref		182
the Netherlands	1.03 (0.74 - 1.45)	0.83	43
Switzerland	0.66 (0.38 - 1.13)	0.13	17
Finland	1.31 (0.78 - 2.17)	0.31	16
Denmark	1.15 (0.70 - 1.88)	0.59	14
Constant	4.58E-5 (3.96E-5 - 5.31E-5)	**<0.001**	
